# Pain relief from combined wound and intraperitoneal local anesthesia for patients who undergo laparoscopic cholecystectomy

**DOI:** 10.1186/1471-2482-14-28

**Published:** 2014-05-12

**Authors:** Chun-Nan Yeh, Chun-Yi Tsai, Chi-Tung Cheng, Shang-Yu Wang, Yu-Yin Liu, Kun-Chun Chiang, Feng-Jen Hsieh, Chih-Chung Lin, Yi-Yin Jan, Miin-Fu Chen

**Affiliations:** 1 Department of Surgery, Chang Gung Memorial Hospital, Chang Gung University, 5, Fu-Hsing Street, Kwei-Shan, Taoyuan, Taiwan; 2 Department of Anesthesiology, Chang Gung Memorial Hospital, Chang Gung University, 5, Fu-Hsing Street, Kwei-Shan, Taoyuan, Taiwan

## Abstract

**Background:**

Laparoscopic cholecystectomy (LC) has become the treatment of choice for gallbladder lesions, but it is not a pain-free procedure. This study explored the pain relief provided by combined wound and intraperitoneal local anesthetic use for patients who are undergoing LC.

**Methods:**

Two-hundred and twenty consecutive patients undergoing LC were categorized into 1 of the following 4 groups: local wound anesthetic after LC either with an intraperitoneal local anesthetic (W + P) (group 1) or without an intraperitoneal local anesthetic (W + NP) (group 2), or no local wound anesthetic after LC either with intraperitoneal local anesthetic (NW + P) (group 3) or without an intraperitoneal local anesthetic (NW + NP) (group 4). A visual analog scale (VAS) was used to assess postoperative pain. The amount of analgesic used and the duration of hospital stay were also recorded.

**Results:**

The VAS was significantly lower immediately after LC for the W + P group than for the NW + NP group (5 vs. 6; p = 0.012). Patients in the W + P group received a lower total amount of meperidine during their hospital stay. They also had the shortest hospital stay after LC, compared to the patients in the other groups.

**Conclusion:**

Combined wound and intraperitoneal local anesthetic use after LC significantly decreased the immediate postoperative pain and may explain the reduced use of meperidine and earlier discharge of patients so treated.

## Background

Since 1987, laparoscopic cholecystectomy (LC) has become the standard procedure for the treatment of gallbladder lesions. Increased experience with this technique has altered some of the previous contraindications for LC such as patients with end-stage renal disease, liver cirrhosis, and severe cardiovascular disease [1-3]. A major benefit of using laparoscopy for upper gastrointestinal surgery is that it avoids an upper abdominal incision. Such incisions hinder postoperative pulmonary rehabilitation, cause surgical wound pain, and increase the total medical cost [4-6]. Laparoscopic cholecystectomy is the treatment of choice for a wide spectrum of gallbladder diseases [7-10]. However, many investigators continue to work to improve postoperative pain control and improve the quality of hospital stays. Many methods have been proposed to improve pain control such as the use of local anesthetics at the trocar site [11,12], intraperitoneal injection of local anesthetics [13-15], decreasing pneumoperitoneum pressure [16-18], and decreasing the number of operative ports [19,20]. The relief of pain resulting from combined wound and intraperitoneal local anesthetics for patients undergoing LC has been evaluated [21]; however, the treatment still needs a full evaluation because of a limited number of patients and inappropriate use of intraperitoneal local anesthetics in the previous study [21]. In this prospective case-controlled study, we clarified the impact of combined wound and intraperitoneal local anesthetic use on pain relief in patients who underwent LC.

## Methods

Between 2009 and 2011, 220 consecutive patients who underwent LC (performed by author C. N. Yeh) at the Department of Surgery in the Chang Gung Memorial Hospital (Taoyung, Taiwan) were included in this prospective case-controlled study. The study participants were adult patients who had been referred for elective LC to treat gallbladder lesions. The study was approved by the local institutional review board of the Chang Gung Memorial Hospital (No. 95-0147B). Patients provided written, informed consent authorizing group allocation and use of anesthetics for pain relief.

### Allocation of the patients

**Figure 1:**
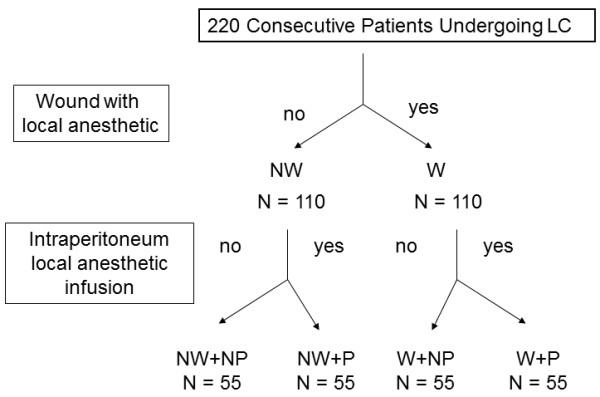
**Algorithm of the group allocation for 220 patients undergoing laparoscopic cholecystectomy.** LC = laparoscopic cholecystectomy; NW + NP = no wound anesthetic and no intraperitoneal anesthetic use; NW + P = no wound anesthetic but intraperitoneal anesthetic use; W + NP = wound anesthetic but no anesthetic use; W + P = wound anesthetic and intraperitoneal anesthetic use.

Each of the consecutive 220 patients was liberally rather than randomly allocated into 1 of 4 groups. The groups included patients who received a local wound anesthetic at the end of LC either with an intraperitoneal local anesthetic (W + P group; n = 55) or without an intraperitoneal local anesthetic (W + NP group; n = 55) and patients who received no local wound anesthetic at the end of LC either with an intraperitoneal local anesthetic (NW + P group; n = 55) or without an intraperitoneal local anesthetic (NW + NP; n = 55) (Figure 1). The diagnostic workup for all patients before LC included a medical history, physical examination, and abdominal ultrasonography. Abdominal computed tomography and/or magnetic resonance cholangiopancreatography might be needed if concurrent biliary tract stone was suspected clinically. Eligible patients had planned LC surgery; had adequate hematological, hepatic, and renal functions; were 20–85 years old; were not pregnant; and provided informed consent. Patients were excluded if they had taken immunosuppressive drug therapy within previous 6 months; had an immunosuppressive condition such as acquired immunodeficiency syndrome, autoimmune disorders, organ transplantation, and radiation therapy; had undergone chemotherapy within the previous 6 months; or had insulin-dependent diabetes mellitus (i.e., type 1 diabetes). Discharge from the hospital was the primary endpoint. Between the groups, we compared the clinical features, laboratory data, operative outcomes, pain score, and the amount of analgesic required. The hospital stay was defined as the number of days between the operation and the actual date of hospital discharge. Surgical mortality was defined as death occurring within 1 month of surgery.

### LC protocol

One author (C. C. Lin), an anesthesiologist, administered general anesthesia to all 220 patients by using the same protocol. Before the start of the cholecystectomy, the LC patients in the W + P group and in the NW + P group received 5 mg/kg of 1.0% levoropivacaine in 200 mL of 0.9% normal saline as an intraperitoneal infusion into the operative field (i.e., infusion after the saline loading test and before carbon dioxide insufflation). Levoropivacaine was administered in 2 separate 100-mL infusions while the patient lay in the Trendelenburg position. The first infusion was administered in the right subdiaphragmatic region and the second infusion was administered in the left subdiaphragmatic region. Patients in the W + NP and NW + NP groups received 200 mL of 0.9% normal saline as the intraperitoneal infusion of the operative field before the start of surgery. All infusion fluid was drained after the completion of the LC. The LC patients in the W + P group and in the W + NP group received a total of 20 mL of 1.0% levoropivacaine at the port sites immediately after wound closure (6 mL at the epigastric port, 6 mL at the umbilical port; and 4 mL at each working port). Patients in the NW + P and NW + NP groups received a total of 20 mL of 0.9% normal saline at the port sites immediately after wound closure (6 mL at the epigastric port, 6 mL at the umbilical port; and 4 mL at each working port). At the end of surgery, local anesthesia or normal saline was applied to the skin, subcutis, fascia, and parietal peritoneum through the port sites.

### Patient monitoring and testing

A visual analog scale (VAS) with a 10-cm vertical score ranged from “no pain” to “worst possible pain”. The VAS was used to assess postoperative pain when the patient awoke in the operating room (approximately 1 h after surgery), at 6 h after surgery, and at 24 h after surgery. The pain score was recorded by 1 of 4 authors (Y. Y. Liu, S. Y. Wang, C. Y. Tsai, or C. N. Yeh) who were blinded to the patient groups. Acetaminophen was used for pain relief in the postoperative period. Meperidine was further used as rescue pain relief if acetaminophen did not work well. Pain intensity was estimated by using the VAS and the amount of analgesics used. Biochemical data, operative time, hospital stay, and perioperative complications were recorded.

### Statistics

All data are presented as the percentage of patients or the mean with the 95% confidence interval or median with interquartile ranges. Numerical data were compared by using analysis of variance (ANOVA) tests between groups. The Scheffe’s test was used as the *post hoc* test for comparing between subgroups. Ordinal scale data were compared with the Kruskal-Wallis test between groups and a *post hoc* test was used for comparing between subgroups. Pearson’s chi square tests and Fisher’s exact tests were used for nominal variables. All statistical analyses were performed by SPSS software (version 10.00) (SPSS, Chicago, IL). Values of p < 0.05 were considered statistically significant.

## Results

### Clinical features, laboratory data, and operative outcomes

Tables 1 and 2 summarize the demographic data and clinical features of the 4 groups of patients with gallbladder lesions who underwent LC (Figure 1). All 4 groups had similar age distribution and gender ratio. The 4 groups displayed no significant difference in the ratio of patients with previous abdominal operations and associated disease and patients without these factors. All 4 groups had similar American Society of Anesthesia (ASA) grades, operation times, and conversion rates. There was no 30-day mortality in any group. No significant difference in laboratory data was noted when between-group comparisons were performed.

**Table 1:** Demographic data of 220 patients who underwent laparoscopic cholecystectomy with or without a local wound anesthetic and with or without an intraperitoneal anesthetic

	**NW + NP (N = 55)**	**NW + P (N = 55)**	**W + NP (N = 55)**	**W + P (N = 55)**	**p**
Age (y)	51.1 ± 15.0	53.3 ± 16.3	50.8 ± 13.5	48.6 ± 14.2	0.505
Gender (M:F)	24:31	26:29	35:20	28:27	0.172
Previous abdominal operation history (+)	11 (20)	11 (20)	15 (27.3)	8 (14.5)	0.429
Associated disease (+)	23 (41.8)	30 (54.5)	21 (38.1)	31 (56.4)	0.142
ASA grade (1:2:3)	18:33:4	14:33:8	28:24:3	17:34:4	0.089
Operation time (min)	84.9 ± 28.7	84.2 ± 22.6	82.4 ± 28.9	90.0 ± 24.1	0.535
Hospital stay (d)	2.3 ± 2.1	2.1 ± 1.0	2.0 ± 0.9	1.6 ± 1.0*	0.039

*ASA* = American Society of Anesthesia; *NW + NP* = no wound anesthetic and no intraperitoneal anesthetic use; *NW + P* = no wound anesthetic but intraperitoneal anesthetic use; *W + NP* = wound anesthetic but no intraperitoneal anesthetic use; *W + P* = wound anesthetic and intraperitoneal anesthetic use.

The data are presented as the number (percentage) or as the mean ± the standard deviation.

*p < 0.05, based on results of the *post hoc* Scheffe test.

**Table 2:** Pain score and analgesic use of 220 patients who underwent laparoscopic cholecystectomy with or without a local wound anesthetic and intraperitoneal anesthetic

	**NW + NP**	**NW + P**	**W + NP**	**W + P**	**p**
Pain analog scale					
1 h post LC	6 (4–8)	5 (3–8)	5 (3.7-6)	5 (3–6)^a^	0.012
6 h post-LC	4 (3–6)	4 (3–5)	4 (2–6)	4 (3–5)	0.330
24 h post-LC	2 (2–3)	2.5 (1–4)	2 (1–4)	2 (2–4)	0.974
Merperidine use (50 mg/pc)					
24 h post-LC	35 (0–62.5)	0 (0–50)	25 (0–50)	0 (0–25)^b^	0.003
Total amount	40 (0–75)	50 (0–50)	35 (0–50)	0 (0–50)	0.500
Acetaminophen requirement (500 mg)					
Total amount	500 (0–2000)	0 (0–625)	500 (0–1000)	0 (0–500)	0.064

*LC* = laparoscopic cholecystectomy; *NW + NP* = no wound anesthetic and no intraperitoneal anesthetic use; *NW + P =* no wound anesthetic but intraperitoneal anesthetic use; *W + NP* = wound anesthetic but no anesthetic use; *W + P =* wound anesthetic and intraperitoneal anesthetic use.

^a^p < 0.05, compared to NW + NP group patients (based on the results of the *post hoc* Kruskal-Wallis test).

^b^p < 0.05, compared to NW + NP group patients (based on the analysis of variance [ANOVA] *post hoc* Scheffe’s test results).

### Evaluation of pain intensity, analgesic requirement, and duration of hospital stay

**Figure 2:**
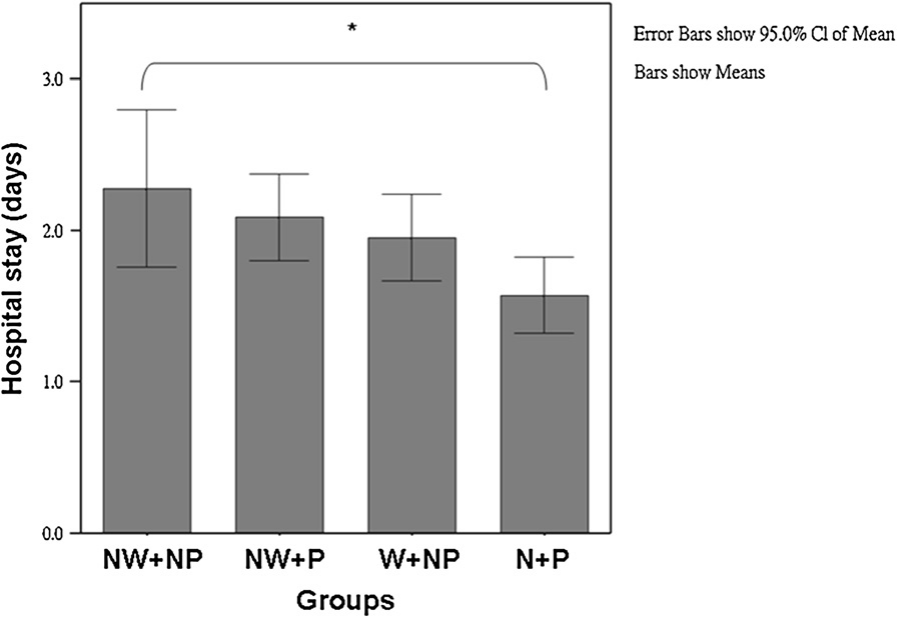
**The duration of the hospital stay of the 4 groups of patients [i.e., patients who received a local anesthetic at the end of laparoscopic cholecystectomy either with an intraperitoneal local anesthetic (W + P group; n = 55) or without an intraperitoneal local anesthetic (W + NP group; n = 55), and patients who received no local wound anesthetic at the end of LC either with an intraperitoneal local anesthetic (NW + P group; n = 55) or without an intraperitoneal local anesthetic (NW + NP group; n = 55)].** *p < 0.05. The W + P patients had significantly shorter hospital stays than the NW + NP patients. All data are presented as the mean with 95% confidence interval standard deviations. NW + NP = no wound anesthetic and no intraperitoneal anesthetic use; NW + P = no wound anesthetic but intraperitoneal anesthetic use; W + NP = wound anesthetic but no anesthetic use; W + P = wound anesthetic and intraperitoneal anesthetic use.

Table 2 shows the comparison of the assessed pain intensity and analgesic requirements of the 4 study groups. All 4 patient groups experienced a gradual reduction in pain after surgery, as evidenced by the pain VAS scores and the need for analgesics. The patients in the NW + NP group had higher VAS scores after LC, compared to patients in the other 3 groups (p = 0.012). By contrast, the VAS was significantly lower in the patients in the W + P group than in the patients in the NW + NP group (5 vs. 6; p < 0.05). The W + P group patients had the lowest VAS scores and therefore required the least amount of meperidine. The W + P group patients also had the shortest hospital stay after LC (1.6 ± 1.0 days), compared to 2.3 ± 2.1 days for the NW + NP group, 2.1 ± 1.0 days for the NW + P group, and 2.0 ± 0.9 days for the W + NP group (p = 0.039) (Figure 2).

## Discussion

Because LC has become the treatment of choice for many gallbladder diseases, postoperative analgesia for LC pain has been evaluated in several prospective studies. Pain after LC is less intense and lasts a shorter time, compared to pain after open cholecystectomy [4-6]. This explains why patients can be discharged within days of LC surgery and can return to their normal daily activities more quickly, compared to open cholecystectomy. However, LC is not a pain-free procedure.

Postoperative pain control is directed at early mobilization, recovery, and discharge. However, pain can play a major role in metabolic and endocrine responses and can impair postoperative pulmonary function. Various methods have been proposed to control LC postoperative pain such as the use of local anesthesia [15], intraperitoneal infiltration of local anesthesia [16], preoperative administration of anti-inflammatory drugs [17], utilizing carbon dioxide (CO_2_) at body temperature, applying intrapleural morphine [18], and the combined use of nonsteroidal anti-inflammatory drug (NSAIDs) and opioids [19].

Several factors have been extensively studied that may moderate pain after laparoscopic surgery such as the use of local anesthesia at the incision sites, intraperitoneal anesthesia, intraoperative irrigation and suction, the number of operative ports, application of pneumoperitoneum, and maintaining pneumoperitoneum pressure. Several mechanisms of postlaparoscopic pain generation have also been proposed such as ruptured blood vessels resulting from the rapid distension of the peritoneum, traumatic nerve traction, release of inflammatory molecules, trauma to the abdominal wall, trauma occurring with the removal of the gallbladder from the abdomen, pneumoperitoneum created by utilizing CO_2_, maintenance of high abdominal pressure, irritation of the phrenic nerve, and application of cold CO_2_[14]. Because pain is multifactorial, no consensus has been reached regarding effective postoperative pain relief for patients who have undergone LC [11]. A number of studies have been conducted in an effort to reduce postoperative pain after this surgery, but the results have varied.

Early studies focused primarily on gynecological procedures [22,23]. However, studies of pain management that are specific to LC have recently been increasing. Recommendations for the treatment of postoperative pain in patients undergoing LC include NSAIDs, local anesthesia injection at the incision sites, opioids, and preoperative steroid use [24,25]. In a previous study [12], we found that applying at the port sites local anesthesia with a ropivacaine infusion at the end of LC significantly decreased immediate postoperative pain. This short-term benefit explains the patients’ reduced need for parenteral analgesics after LC and the earlier discharge of patients who received local anesthesia infusions, compared to patients who did not have this treatment.

Several trials assessing the use of intraperitoneal local anesthetics have yielded conflicting results. The use of intraperitoneal local anesthesia reduces shoulder tip pain. Its value when used in combination with a local anesthetic has been assessed previously [21]; however, there were several drawbacks in that trial. First, the case number in each arm of the trial may have been limited. Second, the timing of the use of an intraperitoneal local anesthetic was inappropriate because of the routine recommendation, based on a literature review and meta-analysis, that an intraperitoneal local anesthetic be used before LC [22,26].

This case–control study is aimed at studying the benefits of combined local anesthetic (i.e., after LC) and intraperitoneal local anesthetic infusions (before LC) for reducing LC postoperative pain. As demonstrated in this study, LC is not a pain-free procedure. Pain remains a prevalent complaint after LC during the early postoperative period. This study clearly showed that pain peaks within the first few hours after the operation, but diminishes greatly by the next day, as demonstrated by the distribution of pain scores and requirement for parenteral analgesics. Incisional pain is reportedly more intense than visceral pain and dominates during the first 48 hours immediately following LC [13]. Therefore, combining the use of analgesics on incisions and in the peritoneum should provide the best pain control. This study confirms our previous finding that routine LC without any local anesthetic is associated with more pain than LC with a local anesthetic. Combined local and intraperitoneal infiltration of levoropivacaine during surgery significantly reduced the immediate pain intensity, reduced the number of patients who required postoperative parenteral analgesics, and reduced the duration of the hospital stay. The combined administration of a local anesthetic and an intraperitoneal anesthetic in patients resulted in a longer period between LC and the need for additional analgesics, compared to patients who did not receive both measures. Furthermore, patients who received combined local and intraperitoneal infiltration of levoropivacaine during surgery required significantly lower doses of analgesics, compared to patients who did not have this treatment. This finding is explained by the lower pain intensity experienced by patients who received the combined local and intraperitoneal infiltration of an anesthetic.

Levoropivacaine is a new long-acting local anesthetic that was developed after evidence of severe bupivacaine-related toxicity emerged. Levoropivacaine contains only the left-isomer of the active chemical, and based on its three-dimensional structure, it has less toxic potential for the central nervous system and heart [27]. Several clinical studies have evaluated its toxicology and clinical profile, but these differences do not appear to affect clinical practice. The relatively low toxic potential of the pure left-isomer supports its use in clinical situations in which the risk of systemic toxicity from overdosing or from unwanted intravascular injection is high (e.g., during an epidural or peripheral nerve block). Adverse effects associated with the use of levoropivacaine for local anesthesia—such as allergic reactions, local tissue toxicity, cardiovascular system toxicity, central nervous system toxicity, and systemic toxicity—are reportedly rare [27]. We did not observe any such adverse effects in our study.

Although our results may support the use of combined wound and intraperitoneal anesthetics for LC patients significantly decreased immediate postoperative pain, there are several limitations inherent in this study. First, this was a prospective case controlled study, and the selection of patients would not be randomized. Although all of the data were collected prospectively and the characteristics of four groups were similar and homogeneous, the selective and recall bias could not be prevented completely. Another obvious limitation of this study is that it was not double-blinded. Hence, biases of the patient’s and surgeon’s attitudes are likely to arise, especially for evaluation of intensity of pain. The advantages of the combined pain relief procedures observed in unblinded patients may lead patients to be more motivated to see the benefit after received “combined pain relief” procedure. Unblinded surgeons may also contribute to the better postoperative recovery as surgeons may take more aggressive attitudes to early discharge after operation. To overcome these limitations, our results should be confirmed by further prospective randomized controlled trials.

## Conclusions

In conclusion, the use of combined wound and intraperitoneal anesthetics for LC patients significantly decreased immediate postoperative pain. This short-term benefit may explain the decreased use of immediate parenteral analgesics and earlier discharge.

## Competing interests

The authors declare that they have no competing interests.

## Authors’ contributions

C-N Y and C-Y T participated equally in data collection and helped to draft the manuscript. C-T C, S-Y W, Y-Y L, F-J H, Y-Y J, and M-F C reviewed the manuscript and provided suggestions. C-C L conducted anesthesia. C-N Y supervised paper editing and the study design. All authors have read and approved the final manuscript.

## Pre-publication history

The pre-publication history for this paper can be accessed here:

http://www.biomedcentral.com/1471-2482/14/28/prepub
